# Location of Tessellations in Ocular Fundus and Their Associations with Optic Disc Tilt, Optic Disc Area, and Axial Length in Young Healthy Eyes

**DOI:** 10.1371/journal.pone.0156842

**Published:** 2016-06-08

**Authors:** Hiroto Terasaki, Takehiro Yamashita, Naoya Yoshihara, Yuya Kii, Minoru Tanaka, Kumiko Nakao, Taiji Sakamoto

**Affiliations:** Department of Ophthalmology, Kagoshima University Graduate School of Medical and Dental Sciences, Kagoshima, Japan; Massachusetts Eye & Ear Infirmary, Harvard Medical School, UNITED STATES

## Abstract

Tessellated fundus is found as common and early-phase characteristic of myopic eyes and their locations are varied among patients. However, the relationship between their locations and morphological parameters of the eyes is still unknown. The purpose is this study is to determine the locations of the tessellations in the ocular fundus of young healthy eyes, and to determine relationships between their locations and morphological parameters of the eyes. This is a prospective observational cross sectional study of 126 eyes of 126 healthy volunteers (mean age 26.0±4.1 years). The eyes were classified into eight groups based on the location of the tessellations; no tessellation, temporal, infra-temporal, inferior, nasal, peripapillary, whole retina, and unclassified tessellations. The degree of optic disc tilt was quantified using a sine curve fitting program on the optical coherence tomographic circle scan images. The correlations between each tessellation location and the axial length, area of the optic disc plus conus (AOC), and optic disc tilt were determined. Forty-four eyes were place in the no tessellation group, 12 eyes in the temporal, 21 eyes in the infra-temporal, 9 eyes in the inferior, 8 eyes in the nasal, 15 eyes in the peripapillary, 11 eyes in the whole, and 6 eyes in the unclassified groups. The differences in the axial lengths between the no tessellation group and the infra-temporal groups were significant. A significant difference was found in the AOC between the no tessellation and the inferior, infra-temporal, and peripapilalry groups. A significant difference was found in the optic disc tilt between the no tessellation and infra-temporal groups (*P*<0.05). The tessellations are located at specific sites in the fundus of young healthy eyes with the infra-temporal location most frequent. It was correlated with some parameters associated with myopia.

## Introduction

High myopia is a common cause of blindness in many countries. It is becoming a more serious problem globally due to changes in the life style and the environment.[1–4] There are many types of changes in myopic eyes, leading to severe visual loss. Posterior staphylomas are one of these changes, and they develop at the end stage of pathologic myopia. Staphylomas are frequently associated with different types of retinochoroidal atrophy [5], and they generally develop after 40-years-of-age. Posterior staphylomas are observed in 90% of the eyes of individuals ≥50-years with pathologic myopia.[6] It is known that a staphyloma can cause a severe stretch of the outer wall of the eye[5] which leads to pathological changes that cannot be detected by conventional ophthalmological examinations in the early stages.

A tessellated fundus is another common characteristic of myopic eyes.[6–8] In healthy human eyes, it has been shown that a tessellated fundus represents an area where the choroid is thin.[9] In pathologically myopic eyes, the retinal pigment epithelium (RPE) and choroid are thinner due to a stretching and expansion of the posterior surface of the eye.[5,10] A tessellated fundus is observed at a younger age than a posterior staphyloma [6–8], and it was recently classified as category 1 in the five categories of myopic maculopathy. They were followed by diffuse chorioretinal atrophy (category 2), patchy atrophy (category 3) and macular atrophy (category 4).[11] Because 10% of the cases in category 1 were reported to progress to category 2 in 12 years [11], some of the tessellations at a younger age might be involved in pathologic retinochoroidal alterations and/or staphylomas in the advanced stage of pathologic myopia.

There are several types of staphylomas present in highly myopic eyes. Curtin classified staphylomas into ten categories based on their location.[8] However to the best of our knowledge, there has not been a study determining the locations of the tessellation in the fundus and whether they are related to other myopic changes. Considering the increasing prevalence of myopia and its association with serious retinochoroidal changes, it is important to know more about tessellated fundi.

Thus, the purpose of this study was to determine the location of the tessellation in the fundus of young healthy subjects and to determine whether the location was correlated with the degree of optic disc tilt, the area of the optic disc including the conus, and the axial length (AL).

## Methods

### Ethics statement

All of the procedures conformed to the tenets of the Declaration of Helsinki, and they were approved by the Ethics Committee of Kagoshima University Hospital. A written informed consent was obtained from all of the subjects. This study was registered with the University Hospital Medical Network (UMIN)-clinical trials registry (No. UMIN000006040).

### Subjects

This was a cross sectional, prospective observational study of 133 eyes of 133 volunteers who were studied between November 1, 2010 and February 20, 2012. Volunteers with no known eye diseases as determined by our examinations were studied, and only the data from the right eyes were statistically analyzed. The eligibility criteria were: age ≥20 years but ≤40 years; eyes normal by slit-lamp biomicroscopy, ophthalmoscopy, and optical coherence tomography (OCT); best-corrected visual acuity (BCVA) ≤0.1 logarithm of the minimum angle of resolution (logMAR) units; and intraocular pressure (IOP) ≤21 mmHg. The exclusion criteria were: eyes with known ocular diseases such as glaucoma and optic disc abnormalities; systemic diseases such as hypertension and diabetes; presence of visual field defects; and history of refractive or intraocular surgery. Nine subjects were excluded; 3 eyes with superior segmental optic disc hypoplasia, 1 eye with glaucoma, 3 eyes with previous laser eye surgery. The data from some of the participants of our earlier study are included in this study.

### Examinations of eyes

All of the eyes had a comprehensive ocular examination including slit-lamp biomicroscopy of the anterior segment, ophthalmoscopy of the ocular fundus, IOP measurements with a pneumotonometer (CT-80, Topcon, Tokyo, Japan), and AL measurements with the AL-2000 ultrasound instrument (TOMEY, Nagoya, Japan). In addition, the refractive error (spherical equivalent) was determined with the Topcon KR8800 autorefractometer/keratometer.

### Classification of lesions in tessellated fundus

**Fig 1:**
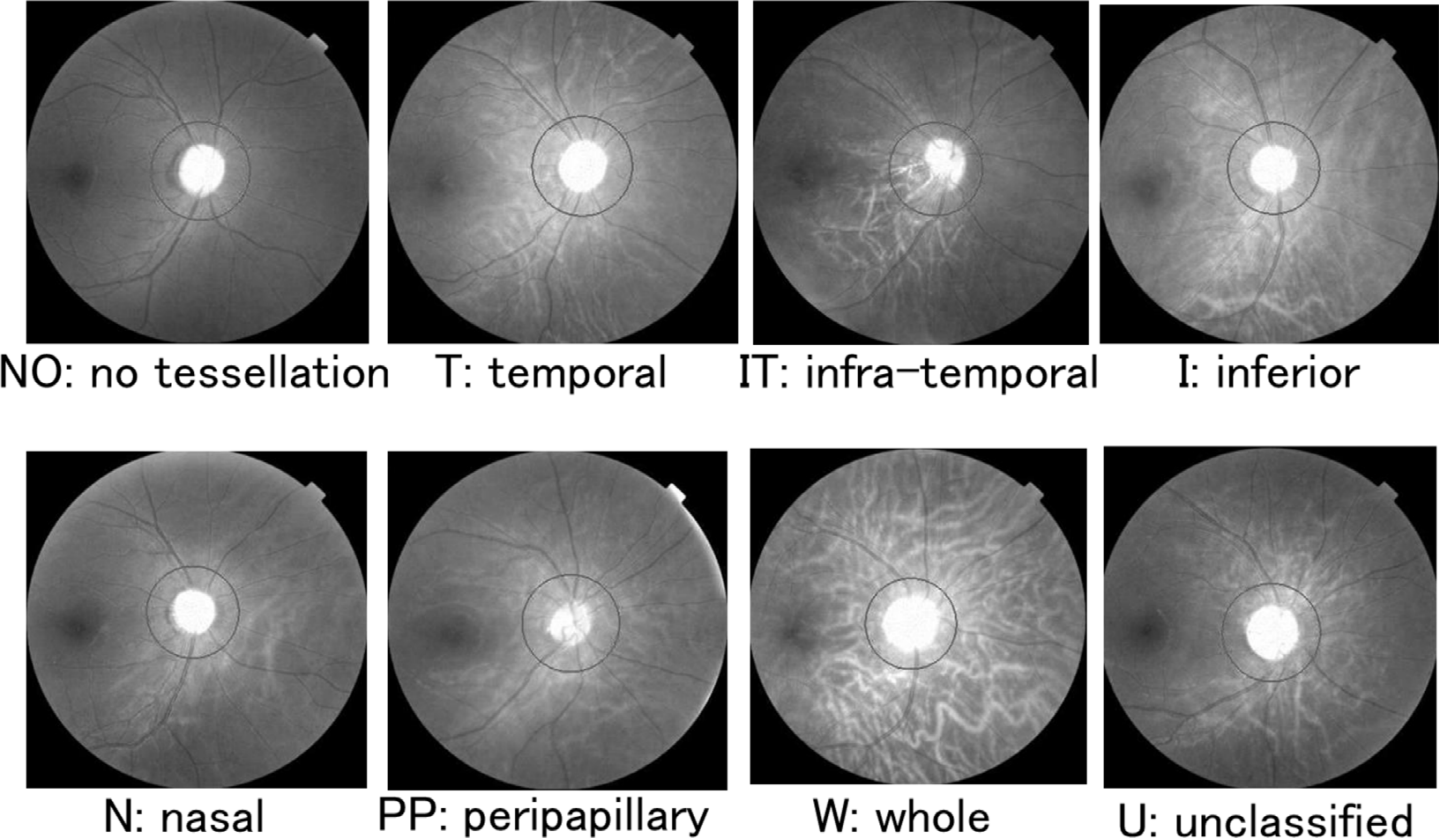
The location of the tessellation using red channel images. Fundus images were exchanged with the split channel command (red) by Image J software to enhance the clarity of the tessellations. The location of the lesions associated with the tessellation was classified based upon Curtin’s classification.

The fundi of all subjects were photographed using the same digital fundus camera with a 45 degree view (TOPCON 3D OCT-1000 MARK II, Topcon). The tessellated fundus lesions were classified into 5 primary types based on Curtin’s classification of the location of the posterior staphylomas, viz., in the posterior pole, macula, peripapillary, nasal, and inferior.[8] We classified the tessellated fundi into corresponding areas such as the whole area, temporal area, peripapillary area, nasal area, and inferior area. We also had an additional location, the intra-temporal area, because several eyes had inferior tessellations with or without foveal lesions. In the end, we classified the tessellated fundus into 8 groups (Fig 1); no tessellation (NO), temporal (T), infra-temporal (IT), inferior (I), nasal (N), peripappilariy (PP), whole (W), and unclassified (U) tessellations. The classifications were made by 2 independent masked examiners. If the raters disagreed, they discussed the reasons for their classification with a third rater, and the final classification was determined by an agreement of at least two of the three graders. If an agreement was not reached, the eye was placed in the unclassified group.

### Measurement of area of optic disc plus conus

The area of the optic disc including the conus was measured in the color fundus photographs with the ImageJ oval section tool. Bennett’s formula was used to correct for the magnification effect of the AL.[12]

### Measurement of optic disc tilt

The method we recently reported was used.[13] Briefly, the degree of optic disc tilt was quantified by fitting a sine curve to the 3.4 mm circle scan, B scan image obtained by Topcon 3D OCT-1000 Mark II. The course of RPE was plotted on the B-scan images manually. The coordinates of each pixel were determined automatically using the ImageJ program. The ‘x’ and ‘y’ coordinates of the B-scan images were converted to a new set of ‘x’ and ‘y’ coordinates with the center of the wave as the origin. Finally, the converted data were fit to a sine wave equation (*y* = *a*×sin(b×x−c)) with the curve fitting program of ImageJ. The value of ‘a’ of the sine curve was defined as the degree of the optic disc tilt.

### Statistical analyses

The intra-rater correlation coefficients of the classification of the tessellated fundus was assessed with the weighted kappa method. The relationships between the location of the tessellations and ocular parameters were determined by Spearman’s rank coefficients of correlation. The Steel-Dwass multiple comparison test was used to determine the significance of the differences in the AL, optic disc tilt, and area of the optic disc plus conus among the groups. All statistical analyses were performed with the statistical programming language R (version 3.0.2, The R Foundation for Statistical Computing, Vienna, Austria). A *P* value of 0.05 was taken to be statistically significant.

## Results

We studied 126 right eyes of 126 individuals. The mean ± standard deviation age was 26.0 ± 4.1 years ranging from 22.0 to 40.0 years, and 85 were men (67.5%). The mean refractive error (spherical equivalent) was -4.71 ± 3.41 diopters (D) with ranging from -14.25 to 4.50 D, and the mean of the AL was 25.43 ± 1.45 mm rainging from 22.3 to 30.4 mm. The mean optic disc tilt was 37.01 ± 17.45 pixels rainging from 4.77 to 80.77 pixels. The mean area of the optic disc plus conus was 2798.89 ± 906.9 pixels rainging from 1478.7 to 7331.4 pixels. The complete dataset is available in S1 Dataset.

### Location of tessellations

The inter-rater agreement in the classification of the location of the tessellations was high with the weighted kappa of 0.87 (*P* <0.001). Tessellations were not found in 44 eyes (34.9%), i.e., the NO group, and found in 82 eyes (65.1%). Among the 82 eyes, 21 eyes with tessellation were placed in the IT group (25.6%), 15 eyes in the PP group (18.3%), 12 eyes in the T group (14.6%), 11 eyes in the W group (13.4%), 9 eyes in the I group (10.9%), 8 eyes in the N group (9.6%), and 6 eyes in the U group (7.3%).

### Comparisons of axial length, optic disc tilt, and area of optic disc plus conus among groups

**Fig 2:**
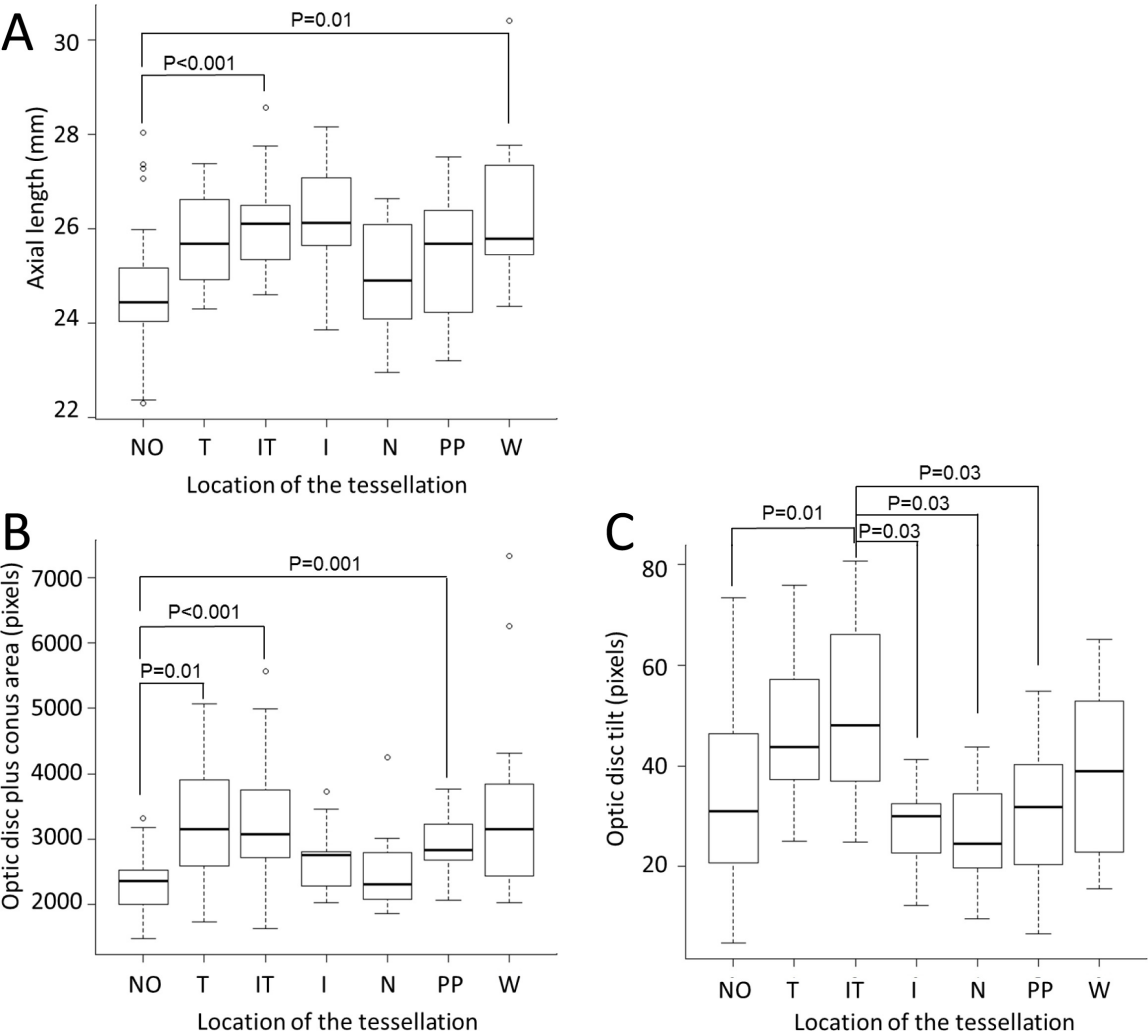
Multiple comparisons of the axial length, area of optic disc plus conus, and optic disc tilt among groups. (A) The AL of the IT group (*P* <0.001) and W group (*P* = 0.01) were significantly longer than that in the NO tessellation group. (B) The area of the optic disc plus conus in the T group (*P* = 0.01), IT group (*P* <0.001), and PP group (*P* = 0.001) were significantly larger than that in the NO tessellation group. (C)The optic disc tilt in the IT group was significantly larger than that in the NO group (*P* = 0.01), the I group (*P* = 0.03), the N group (*P* = 0.03), and the PP group (*P* = 0.03).

We compared ocular findings in eyes with tessellation to eyes without tessellation. The AL of the IT group (*P* <0.001) and W group (*P* = 0.01) were significantly longer than that in the NO tessellation group (Fig 2A). The area of the optic disc plus conus in the T group (*P* = 0.01), IT group (*P* <0.001), and PP group (*P* = 0.001) were significantly larger than that in the NO tessellation group (Fig 2B). The optic disc tilt in the IT group was significantly larger than that in the NO group (*P* = 0.01), the I group (*P* = 0.03), the N group (*P* = 0.03), and the PP group (*P* = 0.03; Fig 2C).

The mean AL of the IT group (*P* <0.001) and the W group (*P* = 0.01) were significantly longer than that of the NO group (Fig 2A). The mean area of the optic disc plus conus in the T group (*P* = 0.01), IT group (*P* <0.001), and PP group (*P* = 0.001) were significantly larger than the area in the NO group (Fig 2B). The optic disc tilt of the IT group was significantly larger than in the NO group (*P* = 0.01), I group (*P* = 0.03), N group (*P* = 0.03), and PP group (*P* = 0.03; Fig 2C).

## Discussion

Our results showed that the tessellations were located in specific regions of the fundus with the infra-temporal location most frequent followed by the para-papillary location. Because we could not find earlier reports on the locations of the tessellations, we compared our results to the locations of posterior staphylomas which are relatively common in eyes with pathologic myopia. If these locations are similar, a closer relationship of the two pathologies might be possible.

Curtin reported that the posterior staphylomas were located in the posterior pole in 55% of the cases, in the macular area in 8.4% of the cases, in the peripapillary area in 1.5% of the cases, in the nasal area in 4.9% of the cases, in the inferior area in 2.6% of the cases, and in compound areas in 27.6% of the cases (subtypes of posterior pole type).[8] For tessellations, the prevalence of the W (whole) type which corresponds to the posterior pole type of staphyloma was 13.4% while Curtin’s posterior pole type was 55%. The prevalence of the T (temporal) type of tessellation was 14.6% which corresponds to the 8.4% of the macular type of posterior staphyloma. Thus, the prevalence of the posterior type of tessellation was lower than the posterior pole type of staphylomas, 13.4% vs 55.0% + 27.6%. In addition, Hayashi et al reported that the prevalence of the posterior pole type of staphyloma was 26.6% and the macular type was 51.7% which are far more prevalent than the other types[7], but similar to that of Curtin’s distribution. The tessellations in this study and the staphylomas in those studies were found mainly in the posterior pole of the eye. However, the frequency of each at a specific location is different, and it was difficult to compare them because of the design of the studies. For example, Hayashi et al investigated hospital-based subjects with visual disturbances while we studied young healthy eyes.

McBien et al followed a large number of normal adults for 2 years and reported that a progression of myopia by an elongation of AL was quite frequent even in adults.[14] So, a remodeling or changing of the structure of eye including localized thinning of the retinochoroidal tissue may occur continuously with increasing age even in adults. Because tessellations become evident at a younger age than posterior staphylomas [7], some of the tessellations at a younger age may progress to posterior staphylomas in eyes with pathological myopia. However, further cohort studies for a longer period are needed to examine this issue.

In general, posterior staphylomas becomes evident after 40-years-of-age.[5] Our results showed that tessellations were most common in the posterior pole as do staphylomas.[7,8] These findings suggest that the localized remodeling of the retinochoroidal tissues leading to a posterior staphyloma may start at a younger age. There is no definite border between a posterior staphyloma and a tessellated fundus, thus these two changes may belong to the same disease spectrum.[7] To obtain a definitive answer on whether tessellations can progress to posterior staphyloma, it will be necessary to perform longitudinal cohort studies.

Our results showed that the AL length was significantly longer in eyes with the infra-temporal type and the whole type of tessellation. Because tessellations are supposedly due to a localized thinning of the RPE/choroid associated with scleral thinning [9], the scleral wall in the tessellated area may be more vulnerable to intraocular pressure than adjacent areas. Thus, this area would easily expand outward. Because the AL is measured as the distance between the cornea and the foveal area, it can be longer in eyes with tessellations within the foveal area than in eyes with tessellations away from the fovea.

Myopic eyes tend to have tilted optic discs [15,16], larger optic disc plus conus area [17,18] than non-myopic eyes. It is clinically important that tessellations were present in the peripapillary area because myopia is a significant factor associated with glaucoma. For example, the degree of optic disc tilt in eyes with infra-temporal tessellation was significantly larger than those in eyes with no tessellation, those with inferior tessellation, nasal tessellation, and peri-papillary tessellations. An optic disc usually tilts laterally, thus the structural changes of the lateral area by tessellations or staphylomas can enhance the optic disc tilting positively, while the changes in the nasal area can do that negatively.

Another issue of tessellated fundi is their relation to the conus.[17,18] A conus is an important myopic change and is supposedly caused by an excessive stretching of the parapapillary area.[19] The present results showed that those eyes with the temporal type, infra-temporal type, and peripallilay type had significantly larger areas of the optic disc plus conus than those with other locations of the tessellations. They are consistent with the idea that both a tessellated fundus and conus are formed by an unusual stretching of the wall of the eye. It is possible that these eyes will further deteriorate into myopic/glaucomatous optic damage. This information should be important in evaluating glaucoma in myopic eyes.

There are several limitations in this study. The sample size was not large enough to detect slight but significant differences between each group. While, the fact that some of the differences reached statistical significance indicates that the findings are sound. Second, we classified the location of tessellations in a subjective way. Although it was classified by two or three independent masked examiners and the inter-rater agreement in the classification of the location of the tessellations was high, new method to classify it objectively would most likely classify it more accurately. Third, this was a cross sectional study, so conclusions on longitudinal effects cannot be made. Fourth, this was a study for young Japanese population. Thus, the present findings may not be applicable to other ethnic groups.

In conclusion, we found that there were specific locations of the tessellations in young healthy eyes. Some of these locations are important sites for making diagnosis of diseases such as myopia and glaucoma. Because their locations were similar to that of posterior staphylomas, the distribution of the locations will be important not only for the diagnosis but also for predicting and preventing the changes in highly myopic eyes.

## Supporting Information

S1 Dataset (XLSX)
